# Micro-Defects-Related Low Cycle Fatigue Mechanical Model of the Nuclear-Grade S30408 Stainless Steel

**DOI:** 10.3390/nano15010071

**Published:** 2025-01-05

**Authors:** Huiping Liu, Mingkun Xiao, Jiannan Hao, Xinjie Ma, Ni Jiang, Qing Peng, Chao Ye

**Affiliations:** 1 Institute of Clean Energy, Yangtze River Delta Research Institute, Northwestern Polytechnical University, Taicang 215400, China; 2 School of Materials Science and Engineering, Northwestern Polytechnical University, Xi’an 710072, China; 3 State Key Laboratory of Nonlinear Mechanics, Institute of Mechanics, Chinese Academy of Sciences, Beijing 100190, China; 4 Center of Materials Science and Optoelectronics Engineering, University of Chinese Academy of Sciences, Beijing 100049, China; 5 Guangdong Aerospace Research Academy, Guangzhou 511458, China

**Keywords:** S304 stainless steel, low cycle fatigue, mechanical model, characteristic defects

## Abstract

Continuous and interrupted low cycle fatigue tests were conducted on nuclear-grade S30408 stainless steel under different stress conditions at room temperature. Vickers hardness testing and microstructure characterization were performed on the fatigue samples with different fatigue states. The evolutionary mechanism of the microstructure defects in materials under fatigue cyclic loading was discussed. The traditional Basquin formula was used to predict the fatigue life of these fatigue samples. At the same time, a quantitative mechanical model related to the characteristic micro-defects parameter KAM and the Vickers hardness (H_v_) was established for the S30408 stainless steel during the low cycle fatigue damage process, and the prediction accuracy of the Vickers hardness is greater than 90%, which is significant and useful for the fatigue life prediction of the 304 stainless steels used in nuclear systems and the safe operation of the reactors.

## 1. Introduction

Due to its excellent heat resistance, corrosion resistance, weldability, and mechanical properties, austenitic 304 stainless steel is widely used in engineering applications [1], such as in automobile parts, ship components, and nuclear power structural components [2,3,4]. In reactors, 304 austenitic stainless steels are normally employed in the core material [5], main pipeline [6], fuel cladding [7], reactor vessel [8], pressurized water reactor [9] and so on. During the service process, the weakening of the metallic materials caused by static-dynamic coupling loading results in fatigue failure, which seriously affects reactor operational safety. Therefore, the fatigue performance and predicted fatigue life of 304 stainless steel have always been important research topics for the long-term safe service of reactor components.

Since the mid-19th century, scholars have conducted extensive research in the field of fatigue damage. The nuclear pressure vessels and nuclear piping components in reactors are typically subjected to relatively high stresses and low failure cycles, which is referred to as low-cycle fatigue [10]. By considering the effect of pre-cycling damage history, a method used to estimate the residual cycles to failure for SUS304 stainless steels was proposed [11]. Furthermore, the application of a small punch test (SPT) was developed and used to evaluate the residual fatigue life of 304 stainless steel [12]. The influence of pressure, temperature, wall thickness, and stagnation time on the fatigue life of 304 stainless steel was investigated using the mechanical fatigue model (MFM) [13]. Moreover, the impact of high-density electrical pulses on the fatigue life of 304 stainless steel was also examined [14]. Additionally, several non-destructive techniques and models utilized to assess the remaining life of 304 stainless steels were developed and verified [15,16,17,18]. Currently, there are two types of widely used damage mechanics models applied to describe the progression of fatigue damage: The first is the Chaboche model (and its various simplified models) [19,20], which is based on the fatigue damage curve directly constructing the damage evolution function. This model is more complex as it has a greater number of parameters. The second is the Lemaitre model [21,22,23,24], which is derived from thermodynamic dissipation theory and is essentially a ductile damage model, where the damage is directly related to the plastic strain.

Overall, there has been little research on the correlation between macroscopic mechanical properties and the microscopic defects of the steel during the fatigue damage service of the 304 stainless steel material in the past few decades. Thus, in this work, commercially purchased nuclear-grade S30408 austenitic stainless steel was employed to perform low-cycle fatigue tests under a range of stress conditions. Samples exhibiting varying cycle numbers in the intermediate unbroken stage were selected for mechanical property testing and microstructural characterization. A quantitative correlation model associating the characteristic mechanical parameters with the characteristic microscopic damage defects was established, which can help researchers to predict the fatigue life of 304 stainless steel more accurately from the microscopic damage mechanism.

## 2. Experiments and Methods

S30408 stainless steel produced by Shanxi Taigang Stainless Steel Co., Ltd., Taiyuan, China, is used as the research material in this research, and its elemental compositions and mechanical properties are shown in Table 1 and Table 2, respectively.

**Figure 1:**
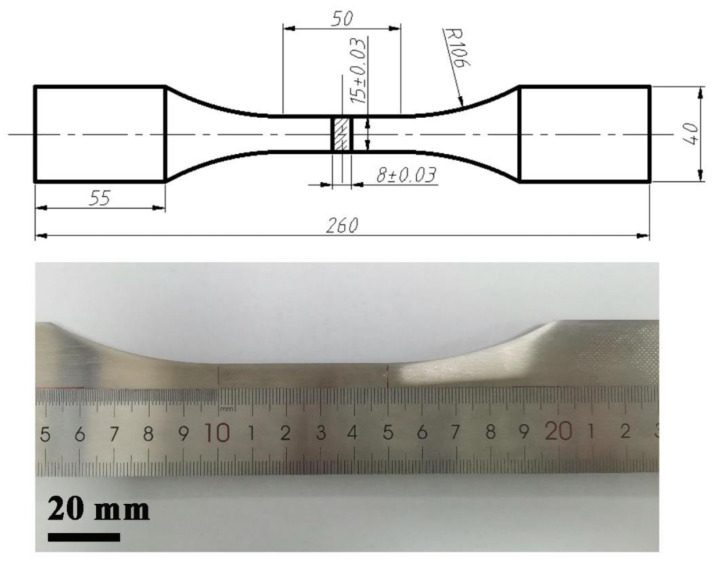
Morphology and size of the S30408 stainless steel fatigue specimens.

First, S30408 stainless steels were processed and polished into fatigue specimens, as shown in Figure 1. Subsequently, in accordance with the standard for axial force control in the fatigue testing of metallic materials” [25], low cycle fatigue experiments—with the constant stress amplitude and stress ratio R = 0.1—were conducted under four stress conditions: 635 MPa, 660 MPa, 700 MPa, and 730 MPa, respectively. In addition, in order to conduct research on the damage changes inside the material during the fatigue processes, according to a series of fatigue life ratios, interrupted low cycle fatigue tests were taken to prepare several fatigue samples with an unbroken state under two selected stress conditions. For the unbroken fatigue samples acquired through interrupted fatigue testing, the stress area in the middle section of these fatigue samples was selected for cutting to obtain block samples with the thickness of about 5 mm. Next, SiC sandpapers, with particle sizes ranging from 500 mesh to 4000 mesh, were used to polish the surface of the samples until it reached a mirror-like state, which was then followed by nanoindentation testing. The Vickers hardness values of the fatigue samples were measured by a Vickers hardness tester. Ten indentations were performed for each sample, and the results were averaged over the ten measurements.

The fracture morphology and grain structure state of the fatigue samples were characterized via a scanning electron microscope (SEM, Zeiss Gemini 460) equipped with electron backscattered diffraction (EBSD) performance. A transmission electron microscope (TEM, FEI talos F200X), equipped with BF (bright field) and DF (dark field) STEM (scanning transmission electron microscopy) mode, was used to observe the microstructural defects inside the fatigue samples, and TEM samples with a thickness less than 100 nm were prepared using the focused ion beam (FIB, FEI Helios G4) lift-out technique.

## 3. Results

### 3.1. Mechanical Properties

**Figure 2:**
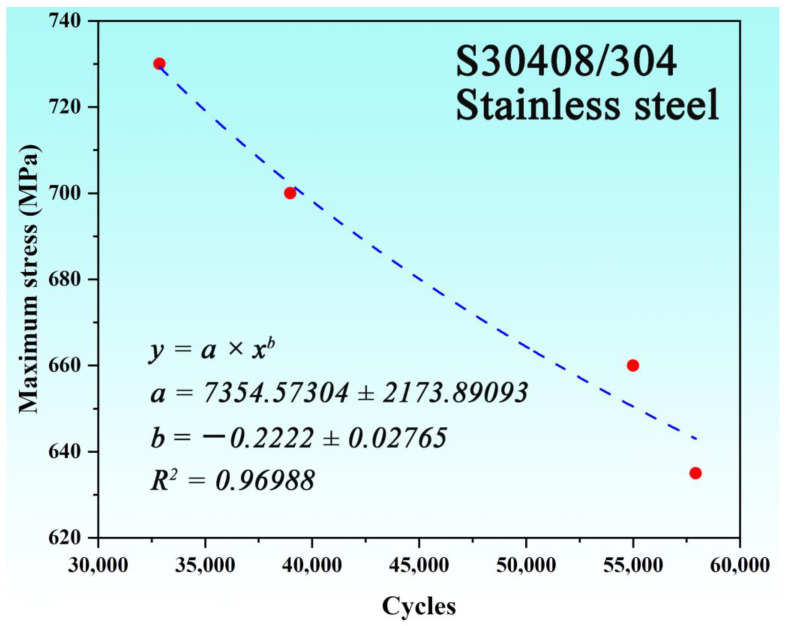
Stress–life curve of the S30408 stainless steel. The discrete red dots represent experimental data points, and the blue dashed line is the fitting curve.

The results of the fatigue tests are shown in Table 3. As the loading stress level gradually increases, the fatigue life of the S30408 stainless steel smooth specimen continues to decline. Based on the low cycle fatigue test parameters controlled by four stress levels and the data of the four low cycle fatigue tests, the maximum stress and fatigue life discrete points were fitted to obtain the stress–life curve, as shown in Figure 2. The quantitative relationship between the maximum stress (*σ_max_*) and fatigue life (*N_f_*) of the smooth S30408 specimens is as follows:

(1) $$\sigma_{max}=7354.57304{\left(N_{f}\right)}^{-0.2222}$$

**Figure 3:**
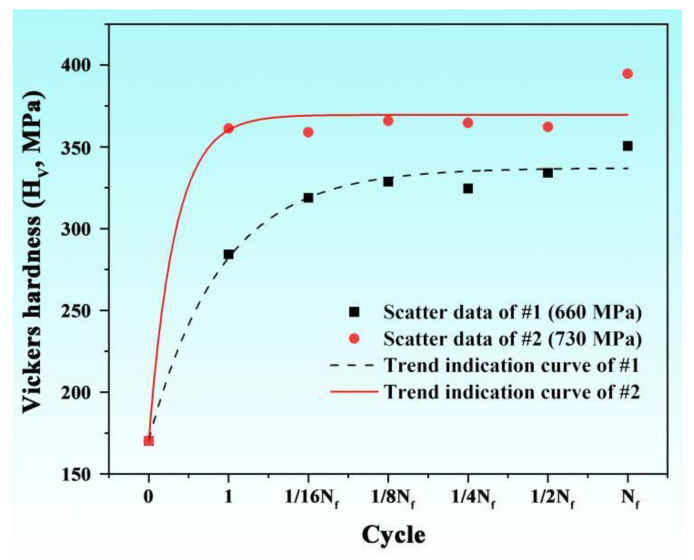
Statistical results of Vickers hardness of the S30408 stainless steel fatigue samples with different cycles.

The main micro-defects caused by fatigue are dislocations, which are always associated with the hardness of the material [26]. The statistical results of the variation in the Vickers hardness with different cycles of the unbroken fatigue samples under 660 MPa and 730 MPa stress fatigue tests are shown in Figure 3. It can clearly be seen from the figure that with the increase in cycles, the hardness of these fatigue samples shows an upward trend, and the increase is mainly concentrated after one cycle. In comparison, the hardness of the fatigue sample under 730 MPa stress is higher than that of the sample under 660 MPa stress at each cycle state.

### 3.2. Microstructure Characterization

**Figure 4:**
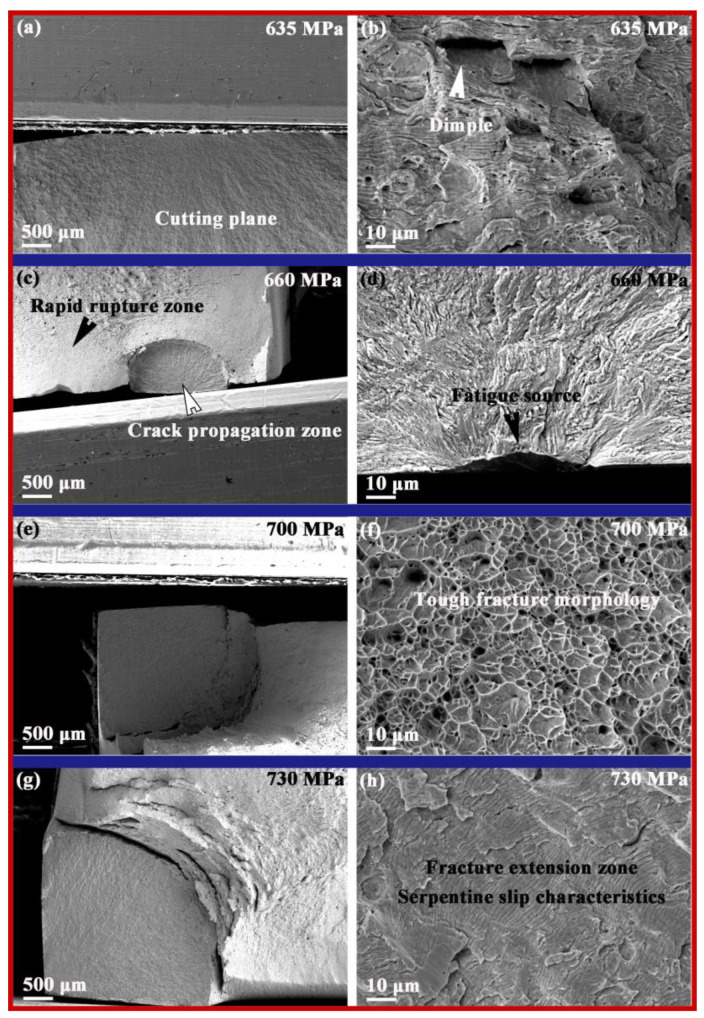
The overall fracture morphologies and local magnified morphologies of S30408 stainless steel fatigue samples under four different stress test conditions: (**a**,**b**) 635 MPa; (**c**,**d**) 660 MPa; (**e**,**f**) 700 MPa; (**g**,**h**) 730 MPa.

The SEM images of the fracture morphologies of the S30408 fatigue samples are shown in Figure 4. No obvious crack source area can be observed on the fracture surface of the fatigue sample under 635 MPa stress (see Figure 4a), but there are obvious ductile dimples on the fracture surface (see Figure 4b). The fracture surface of the fatigue sample under 660 MPa stress clearly shows three different stages of the fatigue fracture process, namely, the slightly depressed fatigue source area on the surface of the fatigue sample shown in Figure 4d, which is generally located in areas with grooves, defects, or stress concentration on the surface and is a prerequisite for the generation of crack sources [27]. The crack source in Figure 4d is located at the center of the surface of the sample in the semi-circular area shown in Figure 4c, which is the crack propagation zone. The cross-section of this area is relatively flat, and the fatigue propagation is perpendicular to the stress direction, producing obvious fatigue arcs, also known as beach lines or shell lines [28,29]. Second, the outer side of the semicircle is referred to as the rapid fracture zone, also known as the instantaneous fracture zone, which is the area where fatigue cracks rapidly propagate to instantaneous fracture. The fracture surface has metal slip marks, and some fracture surfaces have radioactive stripes and shear lips in the instantaneous fracture zone. The fatigue samples under 700 MPa and 730 MPa stress—both fractured from the right angled edge region of the surface (see Figure 4e,g)—and the fracture surfaces show a ductile fracture dimple morphology and a serpentine slip morphology in the port extension region, which is consistent with common fatigue fracture morphology characteristics [30].

**Figure 5:**
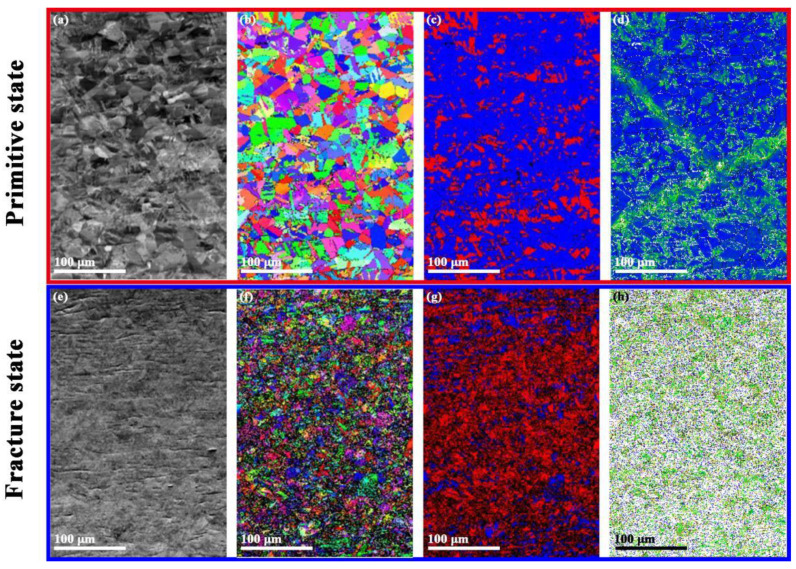
EBSD results of S30408 stainless steel samples under original state and 660 MPa stress fatigue fracture condition: (**a**,**e**) FSD plots; (**b**,**f**) IPF-X diagram; (**c**,**g**) phase distribution diagram; (**d**,**h**) KAM diagram. The different colors in the images represent the grains with different orientations and crystal structures.

In addition, we also carried out EBSD comparative analysis on the original S30408 stainless steel samples and the fatigue fracture samples under 660 MPa stress conditions, as shown in Figure 5 below. Figure 5a,e are FSD diagrams (forescatter detector diagram and surface morphology synthesis); Figure 5b,f are IPF-X reverse pole diagrams (inverse pole figure map, grain orientation-related); Figure 5c,g are phase distribution diagrams (FCC phase and BCC phase distribution); Figure 5d,h are KAM diagrams (Kernel average misorientation is a method used to characterize the local dislocation supporting role angle in EBSD data analysis and is related to the distribution of stress, the dislocation grain boundary, and other defects, indicating the degree of plastic deformation). It can be seen that the shape, size, and orientation distribution of grains can be clearly distinguished from the FSD and IPF images of the original state sample. However, in the fatigue fracture sample, the grain boundaries and morphology distribution are difficult to distinguish. Comparing the phase distribution diagrams (Figure 5c,g), it can be seen that there is a significant increase in BCC phase after fatigue, which may be due to the strain-induced martensitic transformation process of the metastable phase in the original FCC austenite under fatigue-induced stress [31]. In addition, comparing the KAM results (Figure 5c,g) of the original and fractured samples, it can be seen that the fatigue process has severely disrupted the grain boundary orientation inside the sample, resulting in a large number of dislocations.

**Figure 6:**
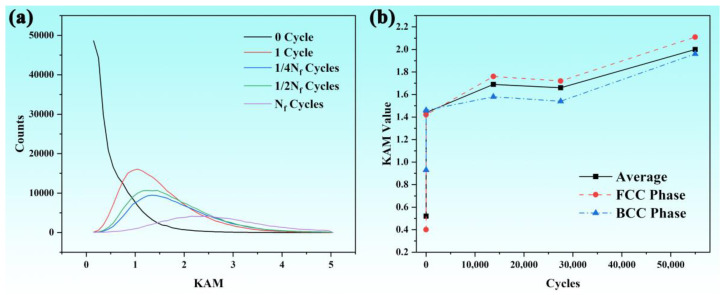
Defect statistics of the S30408 stainless steel fatigue samples under 660 MPa stress conditions: (**a**) KAM count statistics of fatigue fracture samples at different cycles; (**b**) trend chart of KAM variation with cycles.

We conducted a statistical analysis on the KAM values of fatigue samples with different cycles under the 660 MPa stress condition, and the results are shown in Figure 6. Due to severe deformation of the internal grains of the material during the fatigue test, the analytical results of EBSD were not ideal. However, it can still be seen in Figure 6a that as the number of cycles increases, the overall stress inside the grains of the fatigue sample increases significantly, which is mainly due to the contribution of dislocations. And Figure 6b also clearly shows the trend of the KAM value increasing with the increase in cycle times, mainly concentrated after one cycle, which is consistent with the aforementioned trend of the hardness change.

**Figure 7:**
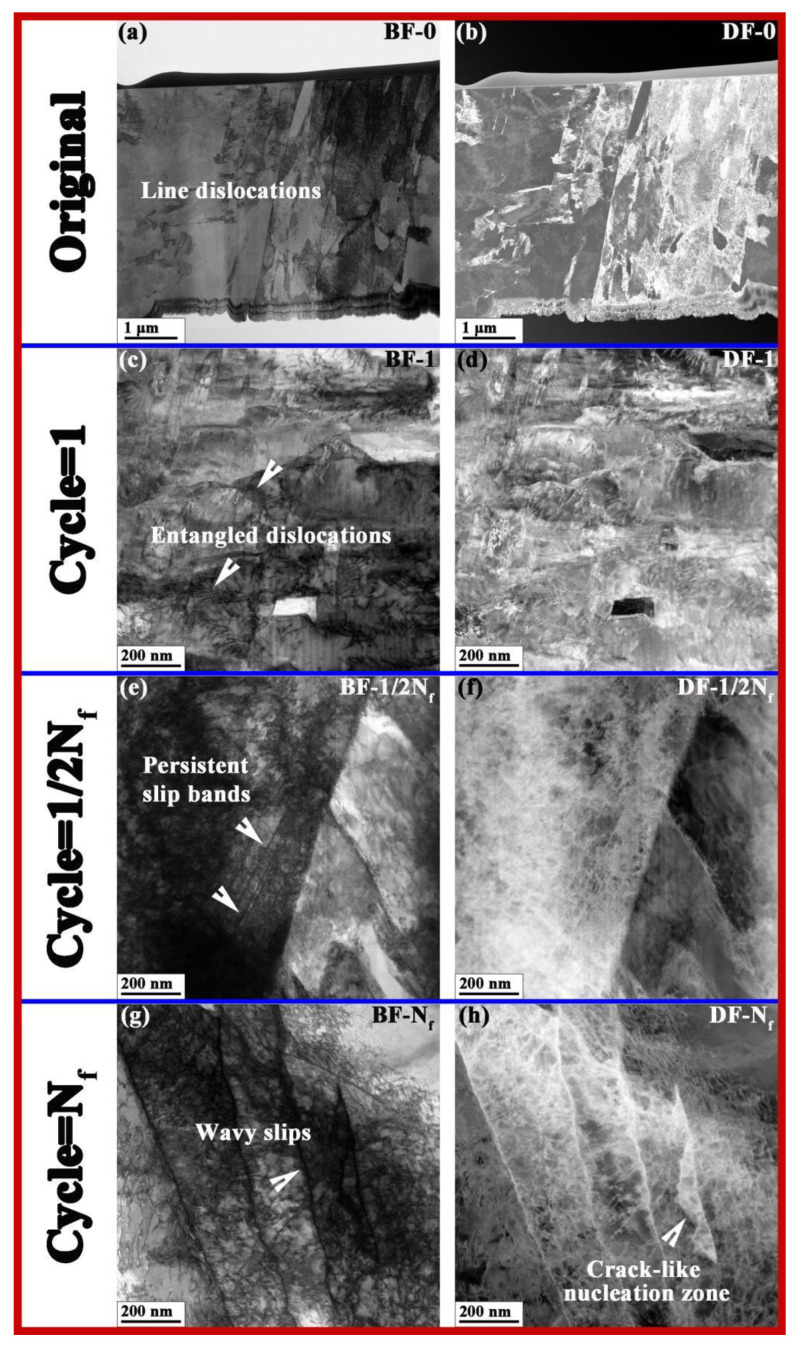
BF and DF STEM images of the S30408 stainless steel fatigue samples under 660 MPa stress condition.

Figure 7 depicts the BF and DF STEM images of the microstructures of fatigue samples with different cycles under the 660MPa stress condition. Under bright-field conditions, dislocation structures exhibit black contrast; whereas under dark-field condition, dislocation structures exhibit white contrast. The interior of the original sample was mainly composed of strip-shaped dislocation lines, as shown in Figure 7a,b. After one cycle, a large number of densely packed dislocations were generated inside the material, and the dislocations began to entangle with each other, as shown in Figure 7c,d. A large number of obvious planar slip bands were generated inside the sample after 1/2*N_f_* cycles, as shown in Figure 7e,f. After *N_f_* cycles of fatigue fracture state, the internal planes of the sample formed wavy slip bands, as shown in Figure 7g,h.

## 4. Discussion

### 4.1. Microstructure

Currently, it is widely believed that macroscopic fatigue failures in metals can be attributed to deformation mechanisms occurring at the micro-nano scale within the internal grains of the material [32,33]. Figure 5 indicates that the S30408 sample underwent severe plastic deformation after fatigue fracture, and the grains size became much smaller as the grain breakage occurred, which is consistent with the KAM statistical results in Figure 6. In addition, the phase distribution diagram results illustrate that most of the face-centered cubic austenitic phases transformed into the body-centered cubic martensitic phases after fatigue damage, as seen in Figure 5c,g. It is generally believed that the austenite grain boundaries and dislocation concentration zones are usually cited as potential nucleation sites for α′ martensite, and the crystallographic structures can be changed under plastic deformation [34,35]. During the process of fatigue damage, the increased nucleation sites accelerated the phase transformation rate, inducing the formation of nanotwins and fine martensite laths. Simultaneously, under the applied stress or strain, the Gibbs free energy of the austenite phase increased. When the energy difference between austenite and α′ martensite reached the threshold for spontaneous transformation ${(∆G}_{RT}^{\gamma \to \alpha^{\prime }}+U^{\prime }\geq {∆G}_{Ms}^{\gamma \to \alpha^{\prime }})$ [36], the α′ martensitic transformation began; the martensitic phase has a higher strength than the austenite phase, and the phase transformation made a certain contribution to the increase in hardness of the S30408 sample during the fatigue process. Additionally, the expansion of residual austenite volume and the plastic deformation induced by the compaction of surrounding soft phases during the phase transformation can also increase the material strength [31,32,37,38].

On the other hand, Figure 7a,e show that a large number of dislocations were generated and entangled after one cycle of fatigue loading, which was induced by plastic deformation [39,40,41]. And the persistent slip bands (PSBs) can clearly be observed in the sample after 1/2*N_f_* cycles of fatigue loading, as shown in Figure 7c,g, which are referred to as the most consequential defect structures with regard to fatigue crack initiation [32]. With the continuation of the fatigue process, the PSBs accumulated and changed into wavy slip bands, a dislocation entanglement structure with larger dimensions, as shown in Figure 7d,h. After *N_f_* cycles of fatigue loading, the PSBs almost occupied the entire sliding region, exceeding the stress threshold for crack formation and ultimately leading to fatigue failure of the material [42,43,44,45]. Overall, the entanglement of dislocations and the martensitic transformation are the main defect evolutions occurring inside the S30408 stainless steels during the fatigue loading processes, both of which can result in an increase in hardness [46,47].

### 4.2. Mechanical Models

According to the Basquin formula [48], during a constant stress amplitude fatigue test, there is a relationship between the stress amplitude and the number of load cycles at which failure occurs, as shown in the formula (2) below.

(2) $$\sigma_{a}=\sigma_{f}^{\prime }{\left(2N_{f}\right)}^{b}$$

where *σ_a_* is the stress amplitude, *σ_f_^′^* is the fatigue strength coefficient, *N_f_* is the number of cycles at constant amplitude load fatigue fracture, and *b* is the fatigue strength index. Meanwhile, the four-point correlation method can be used to determine the fatigue strength index *b* [49].

(3) $$b=-[0.083+0.166log(\frac{\sigma_{f}}{\sigma_{b}})]$$

where *σ_b_* is the tensile strength of the material during static tensile testing. Based on mechanical performance data in Table 2, using following formula (4) [50], which is taken as 1060 MPa, *σ_b_* is taken as 710 MPa, and *σ_f_* is the true fracture strength.

(4) $$\sigma_{f}=\sigma_{b}+350MPa$$

For convenience of calculation, it is generally believed that *σ_f_′ ≈ σ_f_*, so *σ_f_* can be taken as 1060 MPa. Meanwhile, according to formula (3), *b* is calculated as −0.111892. Thus, the fatigue prediction model for S30408 stainless steel is as shown as below.

(5) $$\sigma_{a}=1060{\left(2N_{f}\right)}^{-0.111892}$$

According to the above fatigue model (5), the calculated data and experimental data are shown in Table 4. It can be seen that the theoretical calculation data are in good agreement with the experimental data. The mechanical performance prediction accuracy is greater than 90%, and the maximum error is 7.23%.

During the cyclic loading process of the fatigue load, the reciprocating motion of dislocations can lead to the self-organized growth of long and ordered dislocation structures, thereby causing the nucleation of microcracks in plastic metals. In EBSD characterization, the parameter *KAM* (Kernel average misorientation) can be used to quantitatively calculate the geometrically necessary dislocation density *ρ_GND_*, reflecting the degree of homogenization of plastic deformation. Higher *KAM* values indicate greater plastic deformation or higher defect density in materials. Therefore, it was chosen as a quantitative parameter for the characteristic defects caused by fatigue damage in this research. The average *KAM_ave_* is positively correlated with geometrically necessary dislocation density *ρ_GND_*, satisfying the following relationship [51]:

(6) $$\rho_{GND}=\frac{2{KAM}_{ave}}{\mu b}$$

where b is the dislocation Burger vector, and μ is the EBSD step size.

**Figure 8:**
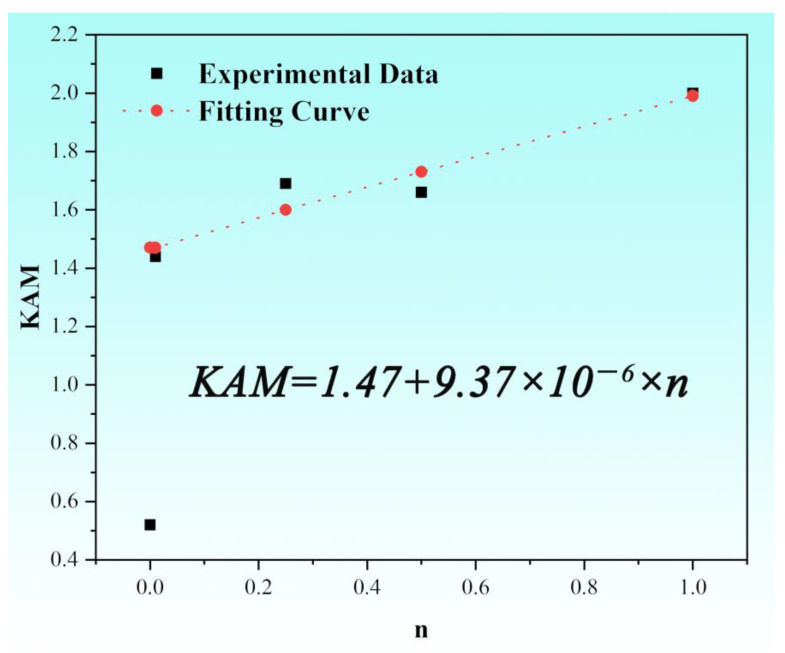
Linear fitting results of the simplified fatigue life *n* and *KAM*.

Different from the ideal laboratory environment, structural components are usually subjected to non-constant fatigue stress in practical engineering process. Therefore, in this study, a parameter, namely, simplified fatigue life *n = N/N_f_* (*N* is the current cycle number; *N_f_* is the fracture cycle number), is defined to evaluate the current fatigue stage and remaining fatigue life of the fatigue samples with a type of percentage. The microstructure characterization results indicate that the dislocation density increased significantly at the early stage of fatigue and then linearly increased. Since the growth rate of dislocations remains basically unchanged when the loading frequency does not change significantly, the relationship between the linear fitting *n* and *KAM* is as follows:

(7) $$KAM=KAM_{0}+kn$$

where *KAM_0_* is the initial *KAM* value (not equal to 0), and $k=9.37\times 10^{-6}$ is the generation rate of dislocations with respect to the simplified fatigue life *n*. The linear fitting results are shown in Figure 8.

**Figure 9:**
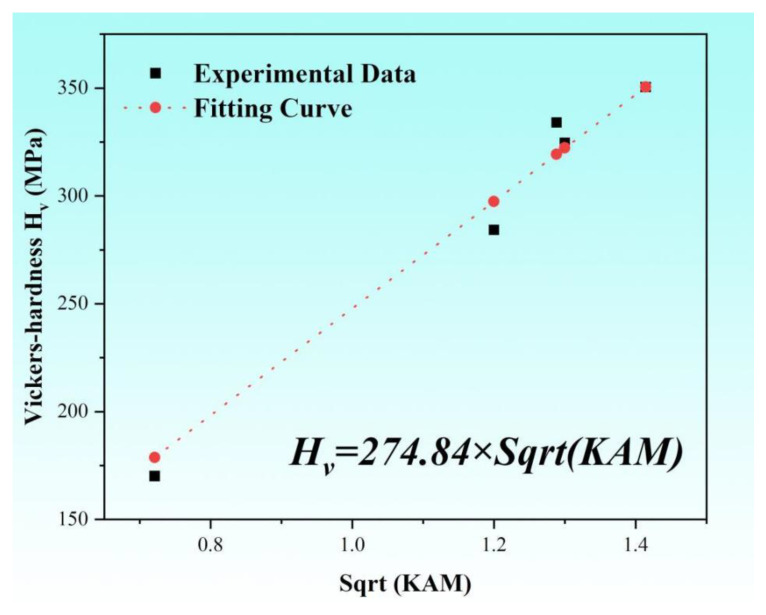
Linear fitting results of the *H_v_* and *KAM*.

The relationship between flow stress σ and dislocation density *ρ_GND_* is as follows [41,52,53]:

(8) $$\sigma =AG\mu \sqrt{\rho_{GND}}$$

where *G* is the shear modulus, and *A* is a constant. The relationship between flow stress and hardness generally follows a linear relationship [54,55]. Therefore, by combining the above formulae, the relationship between Vickers hardness *Hv* and *KAM* can be fitted as in the following equation:

(9) $$H_{V}=B\sqrt{KAM}+C$$

where *B* and *C* are both fitting parameters, and *B* is related to the elastic modulus and dislocation. When *C* is set to 0, the fitting relationship between Vickers hardness *Hv* and *KAM* is shown in Figure 9. The prediction accuracy of Vickers hardness is greater than 90%, and the maximum error is 4.81%.

## 5. Conclusions

Low cycle fatigue tests of nuclear-grade S30408 stainless steels were performed in this work. And a new quantitative mechanical model was established to correlate the characteristic micro-defects induced by fatigue damage with the Vickers hardness. The specific conclusions are listed as follows:

(1) With the increase in fatigue cycle times, plastic deformation led to the continuous generation of dislocations. The reciprocating motion of dislocations resulted in the dislocation entanglement, ultimately leading to material failure due to formation of cracking.

(2) During the low cycle fatigue process, the KAM value and dislocation density increased accordingly, mainly concentrated after one cycle. And the metastable austenite phase underwent a phase transformation to a martensitic phase.

(3) The relationship between fatigue life (N_f_) and loading stress (σ_a_) of S30408 stainless steel satisfies the Basquin equation, and the prediction accuracy of stress amplitude is greater than 90%.

(4) A quantitative model linking fatigue micro characteristic defects and macroscopic mechanical properties was proposed. KAM and Vickers hardness are the characteristic defect parameters and mechanical property parameters, respectively. And the prediction accuracy of Vickers hardness in this model is greater than 90%.

## Figures and Tables

**Table 1:** Chemical composition of S30408 stainless steel (wt.%).

Element	C	Si	Mn	P	S	Cr	Ni	N
Mass Percent	0.04	0.47	1.04	0.030	0.001	18.02	8.04	0.03

**Table 2:** Mechanical properties of S30408 stainless steel.

Brand Number	Yield Strength R_p0.2_ (MPa)	Tensile Strength R_m_ (MPa)	Elongation A5 (%)	Elongation A50 (%)
S30408	281	710	59.0	52

**Table 3:** Results of the fatigue tests.

NO.	Max Stress (MPa)	Interrupted Fatigue State (Cycle)	Fatigue Life (Cycle)	Elongation (mm)
1	635	/	57,928	27
2	660	1, 3437, 6874, 13,749, 27498	54,997	33
3	700	/	38,981	39
4	730	1, 2054, 4108, 8217, 16,435	32,870	42

**Table 4:** The stress amplitude data calculated by the fatigue prediction model and obtained from the fatigue experiments.

No.	σ_max_ (MPa)	N_f_ (Cycle)	Cal. σ_a1_ (Mpa)	Exp. σ_a2_ (Mpa)	Error.(σ_a2_−σ_a1_)/σ_a1_ (%)
1	635	57928	287.5371032	285.75	−0.62%
2	660	54997	289.212464	297	2.69%
3	700	38981	300.5683836	315	4.80%
4	730	32870	306.3580274	328.5	7.23%

## Data Availability

The data presented in this study are available on request from the corresponding authors.

## Abbreviations

*FCC*	Face-centered cubic
*BCC*	Body-centered cubic
*KAM*	Kernel average misorientation
*R_p0.2_*	Yield strength (MPa)
*R_m_*	Tensile strength (MPa)
*A5, A50*	Elongation (%)
*R*	Stress ratio
*σ_max_*	Maximum stress (MPa)
*N_f_*	Fatigue life
*σ_a_*	Stress amplitude (MPa)
*σ_f_^′^*	Fatigue strength coefficient (MPa)
*b*	Fatigue strength index
*σ_f_*	True fracture strength (MPa)
*σ_b_*	Tensile strength (MPa)
*b_d_*	Dislocation burger vector
*μ*	EBSD step size
*n*	Simplified fatigue life
*k*	The generation rate of dislocations with respect to the simplified fatigue life n
*σ*	flow stress
*G*	Shear modulus
*ρ_GND_*	Dislocation density
*H_V_*	Vickers hardness (MPa)
*A*	A constant
*B,C*	Fitting parameters
